# Efficacy and safety of Jin-shui Huan-xian granule for idiopathic pulmonary fibrosis: study protocol for a multicenter, randomized, double-blind, placebo-controlled trial

**DOI:** 10.1186/s13063-022-06684-0

**Published:** 2022-09-02

**Authors:** Shu-guang Yang, Xue-qing Yu, Jian-sheng Li, Yang Xie, Wei Zhang, Chengjun Ban, Jihong Feng, Lei Wu, Xuechao Lu, Limin Zhao, Yong Meng, Miao Zhou, Yong He, Weixian Luo

**Affiliations:** 1 Co-construction Collaborative Innovation Center for Chinese Medicine and Respiratory Diseases by Henan & Education Ministry of People’s Republic of China, Henan University of Chinese Medicine, Zhengzhou, 450046 Henan China; 2 Henan Key Laboratory of Chinese Medicine for Respiratory Disease, Henan University of Chinese Medicine, Jin-shui East Road 156, Zhengzhou, 450046 Henan China; 3 Department of Respiratory Disease, The First Affiliated Hospital of Henan University of Chinese Medicine, Zhengzhou, 450000 Henan Province China; 4 Department of Respiratory Disease, Shanghai Shuguang Hospital, Shanghai University of Chinese Medicine, Shanghai, 200000 China; 5 Department of Respiratory Disease, Dongzhimen Hospital of Beijing University of Traditional Chinese Medicine, Beijing, 100000 China; 6 Department of Respiratory Disease, The Second Affiliated Hospital of Tianjin University of Traditional Chinese Medicine, Tianjin, 300000 China; 7 Department of Respiratory Disease, Hebei Province Hospital of Traditional Chinese Medicine, Shijiazhuang, 050000 Hebei China; 8 Department of Respiratory Disease, Hiser Medical Center of Qingdao, Qingdao, 266000 Shandong China; 9 Department of Respiratory Disease, Henan Provincial People’s Hospital, Zhengzhou, 450000 Henan China; 10 Department of Respiratory Disease, Henan Province Hospital of Traditional Chinese Medicine, Zhengzhou, 450000 Henan China; 11 Department of Respiratory Disease, The Third Affiliated Hospital of Henan University of Chinese Medicine, Zhengzhou, 450000 Henan Province China; 12 Department of Respiratory Disease, Zhengzhou Hospital of Traditional Chinese Medicine, Zhengzhou, 450000 Henan China; 13 Department of Traditional Chinese Medicine, Zhengzhou First People’s Hospital, Zhengzhou, 450000 Henan China

**Keywords:** Idiopathic pulmonary fibrosis, Traditional Chinese medicine, Jin-shui Huan-xian granule, Randomized controlled trial, Study protocol

## Abstract

**Background and rationale:**

Idiopathic pulmonary fibrosis is a critical disease with a poor prognosis. Although different studies have been conducted for the treatment of idiopathic pulmonary fibrosis, limited treatments are available. Jin-shui Huan-xian granule (JHG), which is a Chinese medicine herbal compound, has shown promising efficacy in reducing frequencies of acute exacerbations, improving exercise capacity the quality of life of patients with idiopathic pulmonary fibrosis. This study is to evaluate the efficacy and safety of JHG for IPF.

**Subjects and methods:**

This is a multicenter, randomized, double-blind, placebo-controlled clinical trial. A total of 312 idiopathic pulmonary fibrosis patients will be enrolled and randomly allocated to one of the two groups with 1:1. After a 2-week washout period, 52-week treatment will also be performed for all the patients. Patients in the experimental group and the control group will be given JHG and JHG placebo, respectively. Outcome measures including acute exacerbations, pulmonary function, dyspnea, exercise capacity, and quality of life will be evaluated in this study.

**Discussion:**

Based on our previous study, it is hypothesized that JHG will reduce acute exacerbations; improve exercise capacity, pulmonary function, and quality of life; and delay the disease progression-free. High-level evidence-based support for TCM in IPF will also be obtained in this study.

**Trial registration:**

ClinicalTrials.gov NCT04187690. Register on December 11, 2019

## Introduction

Idiopathic pulmonary fibrosis (IPF) is a progressive interstitial lung disease with poor prognosis and short median survival [1]. The etiology remains not completely clear [2]. With a low prevalence rate, some scholars consider it as a rare disease [3]. In China, it has also been included in the list of rare diseases in 2018. However, different studies show that it has been more common in recent years [4, 5]. After diagnosis, IPF patients will follow a median survival of 2–5 years [5]. Data also shows that IPF patients in Sweden will spend an annual average of $13,975 for hospitalization [6]. In America, a total of 2 billion dollars has been spent on the prevention and treatment of IPF every year [7]. IPF has caused a heavy socio-economic burden.

Although different studies have been performed in recent years, the available treatments remain limited. As reported in the latest international guidelines for IPF, only pirfenidone and nintedanib are recommended in clinical application [8]. They could improve pulmonary function, quality of life, and exercise capacity and delay the disease progression for IPF patients [9–11]. However, the related side effects have also been reported. One study showed that a total of more than 10% of IPF patients taking pirfenidone or nintedanib have discontinued medication permanently due to side effects [12], and their long-term effects are still under debate. Up to now, evidences for other drugs are still insufficient [13–20]. Limited researches have also been performed on non-drug treatment for IPF [21]. However, the levels of the available evidences are also low. It is urgent to conduct further researches on the treatments of IPF [22].

Traditional Chinese medicine (TCM) has different treatments and certain efficacy for respiratory diseases. Chinese herbal medicine (CHM) is the most used measures. According to TCM theory and our previous studies, IPF should be treated according to *feiwei* or *feibi*, and the core TCM pathogenesis includes vital *qi* deficiency, lung collaterals obstruction, phlegm and blood stasis, and accumulation of damage. We have also developed a TCM therapeutic scheme for IPF based on the above studies. Then, the CHM formulation has been optimized to Jin-shui Huan-xian granule (JHG) by clinical application and basic experiments.

JHG is a Chinese medicine compound. Our previous study shows it has a potential treatment effect by inhibiting the differentiation of fibroblasts into myofibroblasts in a pulmonary fibrosis mouse model [23]. We have also performed an exploratory trial to assess the efficacy and safety for IPF patients. The results show that JHG could reduce the frequencies of acute exacerbation and improve lung function and exercise capacity with a reliable safety for IPF patients (to be published). To obtain high-level evidence-based supports, we will conduct a randomized controlled trial to confirm the efficacy and safety of the Jin-shui Huan granule for IPF.

The following contents will be paid attention to in our study: (1) to assess JHG in reducing AEs and improving exercise capacity for IPF, (2) to evaluate the JHG in improving clinical symptoms and quality of life, and (3) safety of JHG will also be assessed.

## Methods/design

### Study design

**Fig. 1:**
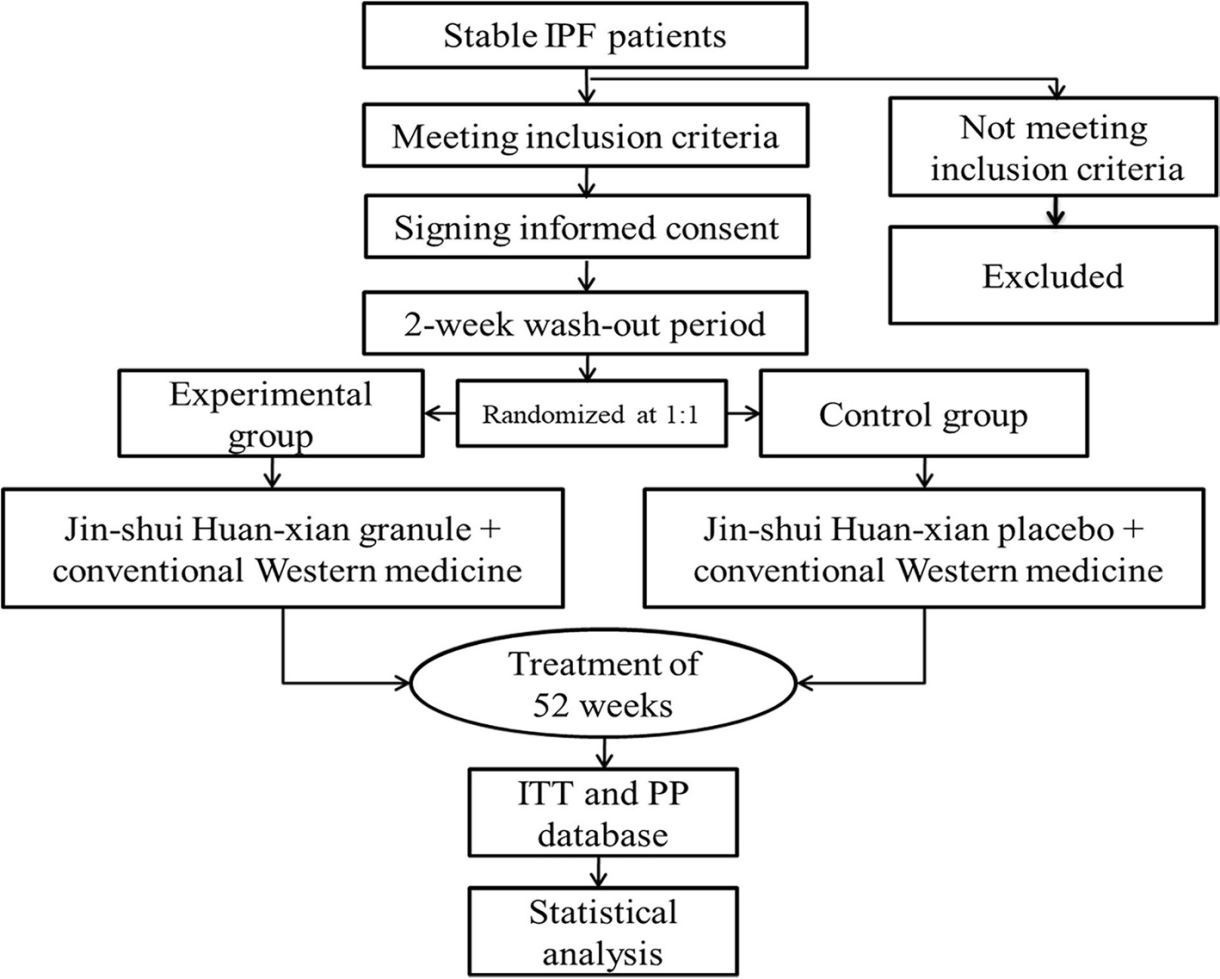
The whole flow chart for this study

This is a multicenter, randomized, double-blind, placebo-controlled clinical trial. The efficacy and safety of JHG for IPF will be assessed in this study. We will recruit 312 stable IPF patients and randomly assign them into the experimental group or the control group with a ratio of 1:1 after a wash-out period of 2 weeks. Then, all the enrolled patients will be given a 52-week treatment of JHG or placebo. The outcomes assessed in this study include frequencies of acute exacerbations, exercise capacity, proportion of progression-free patients, lung function, all-cause mortality, clinical symptoms and signs, dyspnea, health-related quality of life (HRQoL), and adverse events. The protocol follows the Standard Protocol Items for Clinical Trials with Traditional Chinese Medicine 2018: Recommendations, Explanation and Elaboration (SPIRIT-TCM Extension 2018) [24]. The study will also be conducted in accordance with the Declaration of Helsinki (as revised in 2013). The whole study flow chart is found in Fig. 1.

### Patients’ eligibility and criteria

#### Diagnosis criteria of IPF

The diagnosis of IPF will be performed referring to the classification and diagnosis standard of idiopathic interstitial pneumonia published by the American Thoracic Society and European Respiratory Society in 2018 [25]: (1) patients with any other known causes of interstitial lung disease (ILD), such as occupational environment exposure, connective tissue disease, or drugs, should be excluded; (2) for the patients without surgical lung biopsy, high-resolution CT (HRCT) should show a representation of usual interstitial pneumonia (UIP); (3) a specific combination of HRCT and lung biopsy histopathological results should be met for the patients with a surgical lung biopsy; and (4) if necessary, a multi-disciplinary discussion would also be adopted for difficult cases.

#### Diagnostic criteria of TCM syndromes

Because of that, there is a lack of diagnostic criteria for TCM syndromes for IPF; now, the differentiation of TCM syndromes will be referred to the *Diagnostic criteria of TCM syndromes for diffuse interstitial lung disease* [26]. Two independent TCM researchers will perform the syndrome differentiation blindly. If any disagreement appears, the third researcher will be consulted, and a consensus will be reached. The core TCM pathogenesis, which is the accumulation and damage of collaterals due to deficiency of vital qi, will also be taken into account [27]. So, IPF patients with TCM syndromes including lung qi deficiency, lung-kidney qi deficiency, and yin deficiency and inner heat will also be enrolled in this study.

#### Inclusion criteria

Any participant should meet all of the following criteria: (1) meeting the diagnostic criteria of IPF; (2) with ages between 40 and 85 years old; (3) meet the criteria of TCM syndrome differentiation: patterns of lung qi deficiency, lung-kidney qi deficiency, and yin deficiency and inner heat [16]; (4) not participating in any other interventional trial within the previous one month before enrollment; and (5) signing the informed consent form.

#### Exclusion criteria

The patients meeting any of the following criteria will be excluded: (1) pregnant and breastfeeding women or patients with a recent pregnancy plan; (2) delirious, dementia, or with any mental disorder; (3) complicated with severe cardiac insufficiency, liver and kidney diseases, bronchial asthma, chronic obstructive pulmonary disease, tumor, or thoracic deformity; (4) with a condition of limb dysfunction or bedridden; and (5) known to be allergic to any component of the therapeutic drugs.

#### Drop-out criteria

Drop-out criteria have been set up as follows: (1) not willing to continue the trial with any reason and may induce the incompleteness of study data, (2) should not continue to participate in the trial due to allergy or serious adverse events, and (3) patients who are no longer taking the given medication and physical examination and lost in the visit.

#### Eliminated criteria

The enrolled patients will also be withdrawn for any of the following criteria: (1) poor adherence to medication with rates of less than 80% or more than 120%, (2) taking any drug prohibited in this program, and (3) discontinued the given drugs themselves. According to our previous study, the withdrawing rate will be not higher than 20%. All the records for all the eliminated patients will also be preserved.

### Sample size

The calculation formula (*n* = (*u*_*α*_ + *u*_*β*_)^2^(1 + 1/*k*)*σ*^2^/*δ*^2^) based on the comparison of the means of two independent samples has been adopted to calculate the target sample size. *N* and k*n represent the target sample size of the experimental and control groups, respectively. The total variance (*σ*^2^) will be represented by the sample variance (*s*^2^). The formulas *s*^2^ = (*s*_*e*_^2^ + *ks*_*c*_^2^)/(1 + *k*) and *δ* = |‾*x*_*e*_ − ‾*x*_*c*_| have also been adopted. ‾*x*_*e*_ and ‾*x*_*c*_ represent the means of the experimental and control groups, while *s*_*e*_ and *s*_*c*_ represent the standard deviation (SD), respectively. Two-tailed values of *α* and *β* are 0.05 and 0.01, respectively. The ratio of the experimental and control group samples in this trial is 1:1, so the *k* value is 1.

Based on our previous clinical trial (to be published), the frequencies of AEs and 6-min walking distance (6MWD) have been set up as the primary outcomes in this trial. There was a significant difference between the two groups in our previous study for AE. So, the calculation of sample size in this study will refer to 6MWD.

For 6MWD, the mean was 374.38 m in the experimental group and 342.57 m in the control group. The SD was 87.67 m in the experimental group and 81.18 in the control group. The target sample size in each group was 124. Taking a drop-out rate of 20%, the target sample size is 156 in each group, and the total target sample size is 312.

### Ethics, oversight, recruitment, and dissemination

The study has been approved by the Ethical Committees of the First Affiliated Hospital of Henan University of Traditional Chinese Medicine with an identifier (version 2019.07.18.1.0.1.0) 2019HL064-01. Any other revisions of the study protocol will also be submitted to the ethical committee for approval. The whole process of the trial will also be supervised by the Ethical Research Committees of the First Affiliated Hospital of Henan University of Traditional Chinese Medicine. The ethics committee is composed of people with different professions, including non-medical people. The approved protocol will protect the interests of participants. The informed consent will be obtained from all individual participants by the special screener in every research center. Any adverse event will also be submitted to the ethical committees as soon as possible. All the participants will provide written informed consent. Blood and urine samples and exhaled breath condensate will be collected with the participants’ consent for further researches. The authors are accountable for the accuracy of all aspects of the study. Any question related to any part of the work will be investigated and resolved.

Open recruitment for IPF patients will be performed both in outpatient and inpatient. Publishing recruitment advertisements will also be adopted, and the enrolled patients will be observed in 11 research centers in China, which are the First Affiliated Hospital of Henan University of Traditional Chinese Medicine, Shanghai Shuguang Hospital, Dongzhimen Hospital of Beijing University of Traditional Chinese Medicine, the Second Affiliated Hospital of Tianjin University of Traditional Chinese Medicine, Hebei Province Traditional Chinese Medicine Hospital, Qingdao Hici Hospital, Henan Provincial People’s Hospital, Henan Province Hospital of Traditional Chinese Medicine, Third Affiliated Hospital of Henan University of Traditional Chinese Medicine, Zhengzhou Chinese Medicine Hospital, and Zhengzhou First People’s Hospital. All the sub-centers have the qualification and experience in performing TCM trials. Recruitment will last for 12 months from September 2020 or until the target sample is completed. The results of this study will be disseminated to the public in peer-reviewed journals without any patient’s personal information. The raw data will also be available upon request to the authors.

### Randomization and masking

A design of block randomization has been applied to perform the allocation. All the enrolled patients, who meet the inclusion criteria and sign the informed consent, will be randomly assigned to the experimental group or the control group in a ratio of 1:1 after a 2-week wash-out period. The random numbers from 001 to 312 were generated by SAS 9.2 and saved by an independent researcher. The random numbers and corresponding drug codes are the unique identification code of participants. The randomization sequence will be generated in a website program (cloud.medroad.cn) by an independent researcher out of the trial team. After necessary baseline evaluation for each participant who agreed to be enrolled, randomization will be performed. The random number generated by the above website program will be given to a trial team researcher for completing the enrollment. The random sequence will be preserved within opaque, sealed envelopes by an independent researcher for allocation concealment.

All the patients, recruiters, data collectors, outcome assessors, and sponsors will be all blind to the treatment allocation and what each intervention envelope included in the whole process of the trial. In case of emergency, the corresponding random number and treatment allocation will be identified for researchers and supervisors as soon as possible. The randomization design and random numbers are provided by the Jiangsu Famous Medical Technology Co., Ltd. in Nanjing, China.

### Procedures and interventions

Patients in the experimental group will be treated with JHG for 52 weeks, while the control group are treated with a JHG placebo. JHG is a Chinese herbal compound medicine. The compounds of one dosage for JHG are found in Table 1. The placebo used in this study will contain 5% of the same components of JHG. All the JHG and placebo will be produced and packed by Jiang Yin Tian Jiang Pharmaceutical Co. Ltd., Jiangsu, People’s Republic of China, without any difference in the appearance, color, smell, and weight. The drug quality will be tested by the organization with specific qualifications before application and consistent with the required quality standards. Each dosage of JHG granules or placebo has been produced and packed into 4 bags with 10.9 g per bag (Batch number: 2006337). JHG or JHG placebo will be prescribed 2 bags each time, twice a day orally with 5 days on and 2 days off.

**Table 1:** Components of Jin-shui Huan-xian granules

Chinese name	Latin name	Parts of the substances	Amount (g)
Ren Shen	*Radix Ginseng*	Root	6
Shu Di Huang	*Rehm,annia glutinosa Libosch*	Root tuber	12
Mai Dong	*Ophiopogon japonicus*	Root tuber	9
Gua Lou	*Trichosanthes kirilowii Maxim*	Mature fruit	9
Zhe Bei Mu	*Fritillaria thunbergii Miq*	Bulb	9
Mu Dan Pi	*Paeonia Suffruticosa Andrew*	Root bark	9
Zhi Yin Yang Huo	*Epimedium brevicornu Maxim*	Whole herb	6
Chao Bai Guo	*Ginkgo biloba L*	Mature seed	9
Chen Pi	*Citrus reticulata Blanco*	Peel of fruit	9
Zhi Gan Cao	*Glycyrrhiza uralensis Fisch*	Root and rhizome	9

**Fig. 2:**
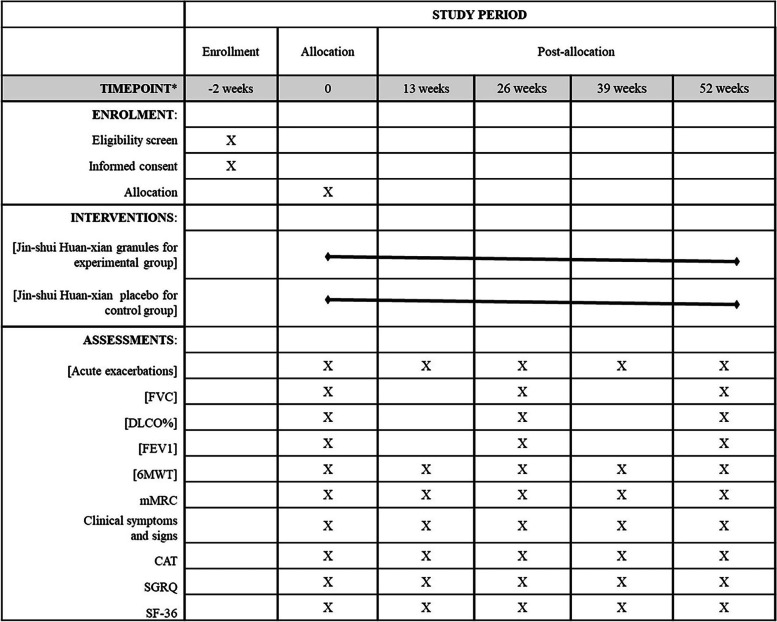
Research flow chart for every participants in the study

At the same time, all the patients will also be treated with conventional modern medicine. Considering the side effects and costs, the application of pirfenidone and nintedanib will not be limited uniformly. During the whole procedure, all the patients will be visited every 13 weeks and receive given physical examinations. Blood and urine samples and exhaled breath condensate will be collected with the participants’ consent for further researches. If patients are under AE, the Chinese medicine granule or placebo will be suspended until the condition becomes stable. To improve the patients’ adherence, the remaining drugs and empty bags will also be returned except for regular telephone follow-up. The whole procedure for each patient is found in Fig. 2.

### Outcome measures

Assessment will be performed by outcome measures at baseline, end of treatment, and given follow-up points. The primary outcomes include frequencies of AEIPF, 6-min walking distance (6MWD), and the proportion of patients with disease progression-free. The secondary outcomes include pulmonary function, all-cause mortality, clinical symptom and signs, dyspnea, and health-related quality of life (HRQoL).

#### Primary outcomes

##### Exacerbations of IPF

The frequencies of acute exacerbations of IPF, which is assessed by AEIPF-related hospitalization, is the primary outcome measure. AEIPF is characterized by significant respiratory deterioration, with an unknown cause within the past 1 month [28]. If the interval between any two AEIPF-related hospitalizations is less than 7 days, they are considered as one AE. The AE will be recorded at every visit, and the total number in the whole process (1-year treatment period) will be calculated. A higher number in a given period indicate the deterioration of IPF and a worse prognosis. A lower number in the experimental group will indicate better efficacy.

##### 6MWD

6MWD is adopted to assess the exercise capacity of IPF patients. It is evaluated by the distance an IPF patient can walk as fast as he/she can in 6 min. A longer distance indicates better exercise capacity for IPF patients.

##### Proportion of disease progression-free survivals

The endpoints of free disease progression in this study include any of the following events [29]: forced vital capacity (FVC) decreased by 10%, diffusing capacity percentage of the predicted value (DLCO%) by 15% compared to baseline, patient death, and lung transplantation. At the end of treatment, the proportion of the patients without any of the above endpoints will be calculated.

#### Secondary outcomes

##### Pulmonary function

FVC and DLCO% will be examined at baseline and at the end of the treatment. A positive change will indicate the improvement of pulmonary function.

##### All-cause mortality

Any death for IPF patients in this study with any cause will be recorded and counted. All-cause mortality will be calculated at the end of the treatment in each group. A lower number in the experimental group indicate a better efficacy for the intervention in improving prognosis.

##### Clinical symptoms and signs

Assessment will be performed by a clinical symptom assessment questionnaire. The clinical symptoms to be evaluated in this study include cough, expectoration, chest tightness, shortness of breath, wheezing, and cyanosis. A score of 0–3 will be given to every symptom or sign with a higher score indicating a worse condition.

##### Dyspnea

Dyspnea will be assessed by the Modified Medical Research Council (mMRC) scores set up by the American Thoracic Society [30]. A score of 0–4 will be given according to the degree of immediate dyspnea. A higher score indicates worse dyspnea.

##### Health-related quality of life

HRQoL will be assessed by the COPD Assessment Test (CAT), St. George’s Respiratory Questionnaire (SGRQ), 36-Item Short-Form Health Survey (SF-36), and A Tool to Assess Quality of life in IPF (ATAQ-IPF), with a higher value indicating a better HRQoL for SF-36 and a worse HRQoL for CAT, SGRQ, and ATAQ-IPF. CAT [31], which includes 8 items with a value of 0 to 5 for each item, is a self-assessment questionnaire commonly used in COPD evaluation. Because of the similarity of the assessment symptoms, it was adopted in this study. SF-36 [32], which includes 36 items in 8 domains, is a universal scale for the assessment of health impairment in a wide range of field. SGRQ [33], with 50 items in 3 domains, is widely adopted to assess health impairment for respiratory diseases. The total scores of SGRQ and SF-36 will be counted by weighing of each item. ATAQ-IPF is an IPF-specific questionnaire to assess the HRQoL of IPF patients. There are 13 domains and 74 items with a score of 1–5 for each item in ATAQ-IPF [34]. All the questionnaires will be completed by the patients themselves. Patients should choose an option which is most consistent with their current condition for each item. If necessary, the researchers could provide help. Then, the researchers complete the scoring.

#### Safety outcomes

We will also evaluate the safety of interventions in this study. Safety outcome measures, including routine blood, urine, and stool tests; liver and kidney function tests; and electrocardiography, will be examined. Any adverse events will also be recorded and managed according to the degrees of damage at any time they occur during the treatment period. Then, we will also report to the ethics committee for further management. Based on our previous studies, the possible adverse events are transient gastrointestinal discomfort without the need for intervention.

Pulmonary function, routine blood, urine and stool tests, liver and kidney function tests, and electrocardiography will be tested and recorded at baseline (week 0), at 6 months after treatment (week 26), and at the end of the treatment (week 52). 6MWT, clinical symptoms and signs, MMRC, CAT, SF-36, SGRQ, and ATAQ-IPF will be completed and recorded at week 0, week 13, week 26, week 39, and week 52 in the process of trial.

##### Quality control

To help improve the quality of research, different quality control measures will be adopted throughout the whole process. Before the study, a research team including clinicians, statisticians, ethicists, pathologists, and radiologists has been set up to discuss and form the study protocol, and standard operating procedures (SOPs) for each step of the trial, such as registration, recruitment, and visit for all the enrolled patients, have also been formulated by the research team to ensure the accuracy and completeness of the obtained data in the trial. Every researcher in each center will be trained on the SOPs. The research procedures, completion and normalization of case report forms (CRFs) in each center will also be examined periodically by independent researchers, which is another important means to help improve research quality.

##### Data management and statistical analysis plan

Statistical analysis will be conducted both in the intent-to-treat (ITT) and pre-protocol (PP) data. ITT analysis will be applied to analyze the safety data. All the data from any included patients will be analyzed regardless of their withdrawal from the trial. The PP analysis will be adopted to assess the efficacy of the interventions. If data permit, sub-group analysis will also be performed to help clarify the therapeutic effect of interventions.

All the *P* values will be set as two-tailed with a significant level of 0.05. Measurement data will be shown with mean ± SD, median, and inter-quartile range. Count data will be presented by frequency or composition ratio. Analysis of variance (ANOVA) will be used to test for the differences in continuous variables, with the chi-square test for categorical variables. If necessary, the paired *t*-test will be applied to intra-group comparison data with normal distribution and homogeneous variance, and the Wilcoxon signed-rank sum test will be used for non-normal distribution or uneven variance. Repeated-measures ANOVA will be used to test the continuous response variables that are measured at more than two time points for the same patient, such as 6MWT, clinical symptoms and signs, mMRC, and HRQoL. To improve the quality of data, all the data management and statistical analysis will be conducted by the Jiangsu Famous Medical Technology Co., Ltd. in Nanjing, China.

## Anticipated results

Based on our previous study, it is hypothesized that JHG will reduce the frequencies of acute exacerbations; improve exercise capacity, pulmonary function, and quality of life; and delay the disease progression-free. High-level evidence-based support for traditional Chinese medicine in idiopathic pulmonary fibrosis will also be obtained in this study.

## Discussion

TCM has a long history and certain efficacy in the treatment of respiratory diseases. However, there was still no consensus for disease name and core pathogenesis of IPF in TCM, which also limits the clinical application. We have also conducted abundant researches in TCM for IPF. Based on the summary of the literatures and clinical experience, we hold that IPF should be treated by referring to *feiwei* or *feibi* in TCM [35].

We have also conducted an exploratory trial to evaluate the efficacy and safety of TCM treatment based on syndrome differentiation preliminarily. The results preliminarily clarified the efficacy and safety of TCM treatment based on syndrome differentiation and provide evidence-based support for TCM in IPF. However, the interventions were given according to the different patterns of syndrome differentiation. It may be difficult for clinical application, especially for non-TCM doctors. For TCM, treatment based on syndrome differentiation has been the critical method for thousands of years. In general, TCM doctors will discriminate the different patterns of syndromes first according to the clinical performance and characteristics. However, clinical performance and characteristics are atypical, which would limit TCM syndrome differentiation, and the sample size is small, which will limit the level of evidence. Therefore, to obtain high-level evidence-based support and apply it conveniently, further confirmatory study for core TCM pathogenesis and Chinese medicine is urgent. As we also know, this is the first multi-centers large-sample randomized controlled trial to clarify the efficacy and safety of Chinese herbal medicine.

In recent years, different studies have shown the efficacy and safety of TCM for IPF [36, 37]. Most of them were small-sample studies with a deficiency of standard design and research procedures for high-level evidences. Evidence-based supports of TCM for IPF have not been well-presented. The clinical application has also been limited. In view of these deficiencies, we adopted a design of standard RCT based on the discussion and conclusion of the previous studies. The international standard study procedure, such as trial registration, patient enrollment, and follow-up, has also been adopted. To further improve the quality of the study, related SOPs have also been formulated. In the future study process, the implementation of SOPs will be examined and discussed periodically. If necessary, we will adjust the part of unsuitable contents according to the actual state. We believe that this study will also provide some references for the TCM standard researches.

Our previous study has revealed the possible active components and potential targets for JHG in the treatment of IPF [38]. A total of 136 compounds from JHG and 265 potential targets have been found. The compounds could exert a synergistic effect by regulating these similar targets probably. JHG could also ameliorate pathological changes and collagen deposition in bleomycin-induced pulmonary fibrosis rats [23], and inhibited NADPH oxidase 4 (NOX4) levels and increased the nuclear factor erythroid 2-related factor 2 (Nrf2) in lung tissues could also be found. In vitro, transforming growth factor β1 (TGF-β1)-induced differentiation of fibroblasts could also be significantly inhibited by JHG. So, we concluded that JHG may show long-term effects in bleomycin-induced pulmonary fibrosis rats. The clinical efficacy of JHG will also be confirmed by the results of this study.

However, there are still some limitations to this study. This study aims to the core TCM pathogenesis and Chinese medicine for IPF. TCM treatment based on syndrome differentiation is not taken into account. The actual advantages of TCM, such as individualized treatment, will be discounted. However, it is unavoidable under the current research conditions. Combined with studies with other design types, such as cohort studies, the advantages of TCM may be presented better at different levels.

Based on our previous research, this study will confirm the hypothesis that JHG could reduce acute exacerbation, improve exercise capacity, delay disease progression, and improve pulmonary function and quality of life for IPF patients with sound safety. The results will provide high-level evidence-based supports for TCM in the treatment of IPF. The study design will also provide critical references for TCM studies on core pathogenesis.

## Trial status

At the moment of manuscript submission, 70 IPF patients have been enrolled in this study and recruitment is in progress.

## Data Availability

Data availability is not applicable for this article as no datasets have been generated or analyzed during the current stage.

## Abbreviations

6MWD: Six-minute walking distance
AE: Acute exacerbation
ANOVA: Analysis of variance
ATAQ-IPF: A Tool to Assess Quality of life in IPF
CAT: COPD assessment test
DLCO%: Diffusing capacity percentage of the predicted value
FVC: Forced vital capacity
HRQoL: Health-related quality of life
ILD: Interstitial lung disease
IPF: Idiopathic pulmonary fibrosis
JHG: Jin-shui Huan-xian granule
mMRC: Modified Medical Research Council
RCT: Randomized controlled trial
SF-36: 36-Item Short-Form Health Survey
SGRQ: St. George’s Respiratory Questionnaire
SOPs: Standard operating procedures
TCM: Traditional Chinese medicine
